# State Policies to Support Family Caregiving

**DOI:** 10.1093/geroni/igaf122.1661

**Published:** 2025-12-31

**Authors:** Rita Choula

**Affiliations:** AARP, Washington, District of Columbia, United States

## Abstract

Family and friends provide 64% of caregiving hours in the US. While uncompensated, the value of this care is estimated to be $600 billion per year. This presentation will share experience of AARP in developing policies to support family caregivers, with example form selected promising state and local programs.

